# Supplementary material for: SAR131675, a VEGRF3 Inhibitor, Modulates the Immune Response and Reduces the Growth of Colorectal Cancer Liver Metastasis
Source: Cancers (Basel). 2022 May 31;14(11):2715. doi: 10.3390/cancers14112715 (PMC9179346; doi:10.3390/cancers14112715)
Supplement: Supplementary file 1 [file cancers-14-02715-s001.zip › Figure S1.pdf]

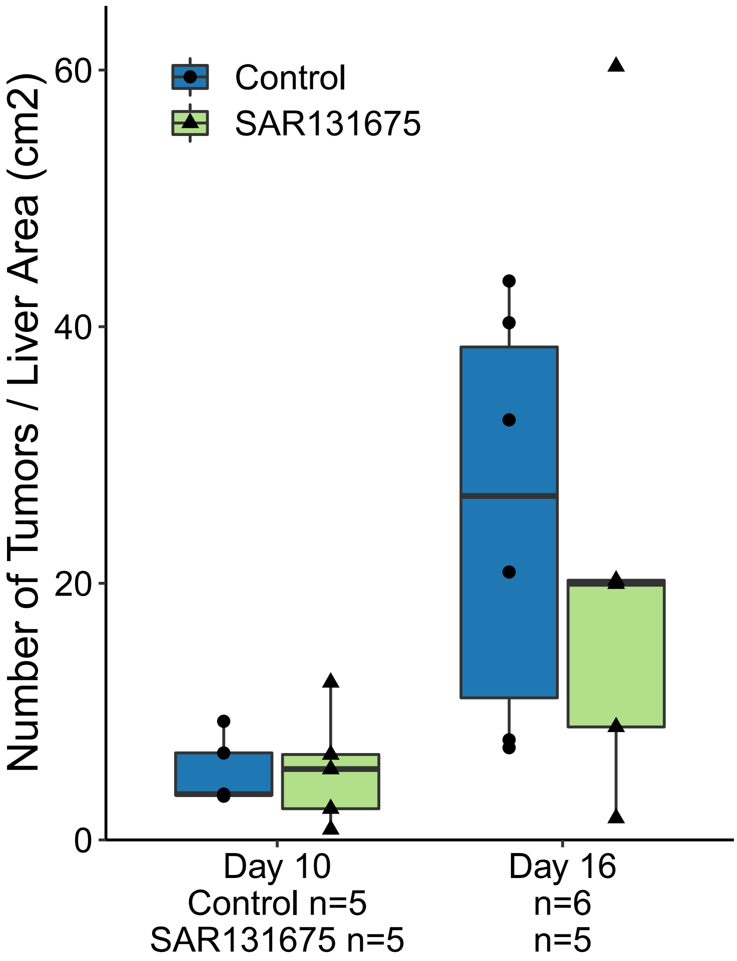

Figure S1 Number of tumours per area (cm2) for each time point.
Up to 5 non-consecutive sections per animal were stained for hematoxylin and eosin (H&E). The number of tumor foci were counted for the whole tumor area (cm2) calculated.
