# Supplementary material for: SAR131675, a VEGRF3 Inhibitor, Modulates the Immune Response and Reduces the Growth of Colorectal Cancer Liver Metastasis
Source: Cancers (Basel). 2022 May 31;14(11):2715. doi: 10.3390/cancers14112715 (PMC9179346; doi:10.3390/cancers14112715)
Supplement: Supplementary file 1 [file cancers-14-02715-s001.zip › Figure S3.pdf]

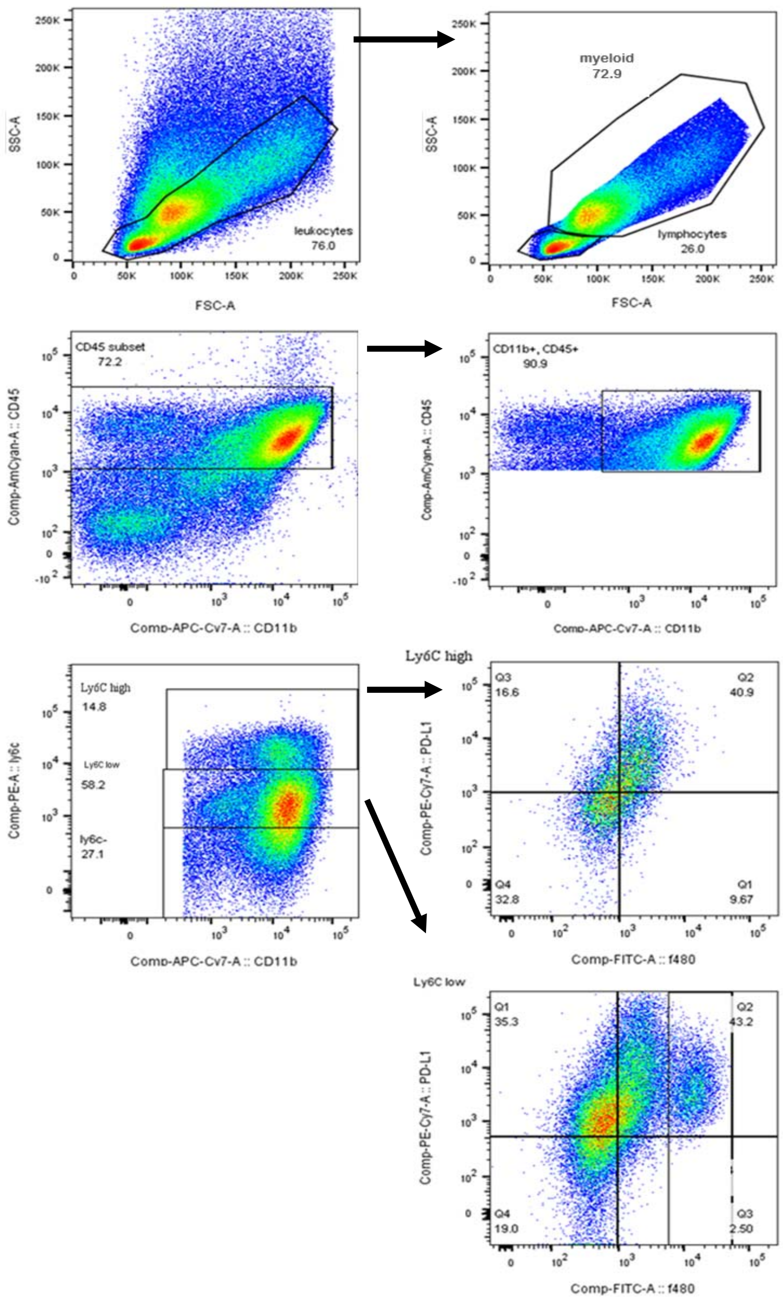

Figure S3 Gating strategy used to identify MDSC and macrophages.

Sequential exclusion gating was used to remove doublet cells and dead (DAPI negative) from the analysis. Size and granularity was used to select large granular cells (gate labelled myeloid). $CD45^{+}$ leukocytes and $CD11b^{+}$ myeloid cells were gated and analysed for the expression of Ly6C. Ly6C expression was divided into Ly6C high, Ly6C low and Ly6C negative populations and analysed for F4/80 expression and PD-L1.
