# Supplementary material for: SAR131675, a VEGRF3 Inhibitor, Modulates the Immune Response and Reduces the Growth of Colorectal Cancer Liver Metastasis
Source: Cancers (Basel). 2022 May 31;14(11):2715. doi: 10.3390/cancers14112715 (PMC9179346; doi:10.3390/cancers14112715)
Supplement: Supplementary file 1 [file cancers-14-02715-s001.zip › Figure S4.pdf]

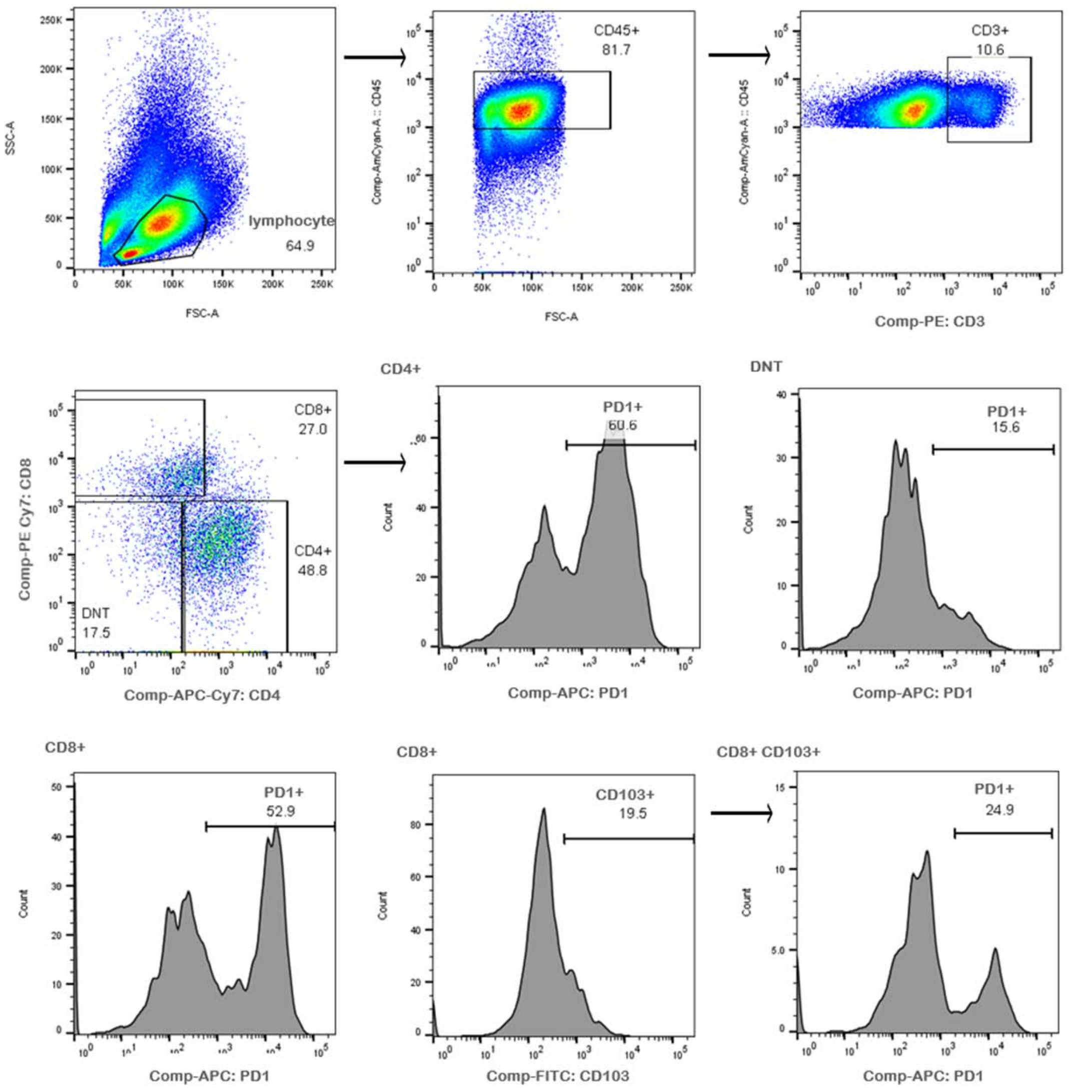

Figure S4 Gating strategy used to identify T-cell populations.

Sequential exclusion gates removed dead (DAPI-) cells, doublet cells and auto fluorescent cells. Size and granularity was used to select both small and larger lymphocytes. CD45+ and CD3+ cells were selected and of these the CD8+, CD4+ and double negative phenotype analysed for CD103 and PD1 expression.
