# Supplementary material for: SAR131675, a VEGRF3 Inhibitor, Modulates the Immune Response and Reduces the Growth of Colorectal Cancer Liver Metastasis
Source: Cancers (Basel). 2022 May 31;14(11):2715. doi: 10.3390/cancers14112715 (PMC9179346; doi:10.3390/cancers14112715)
Supplement: Supplementary file 1 [file cancers-14-02715-s001.zip › Figure S6.pdf]

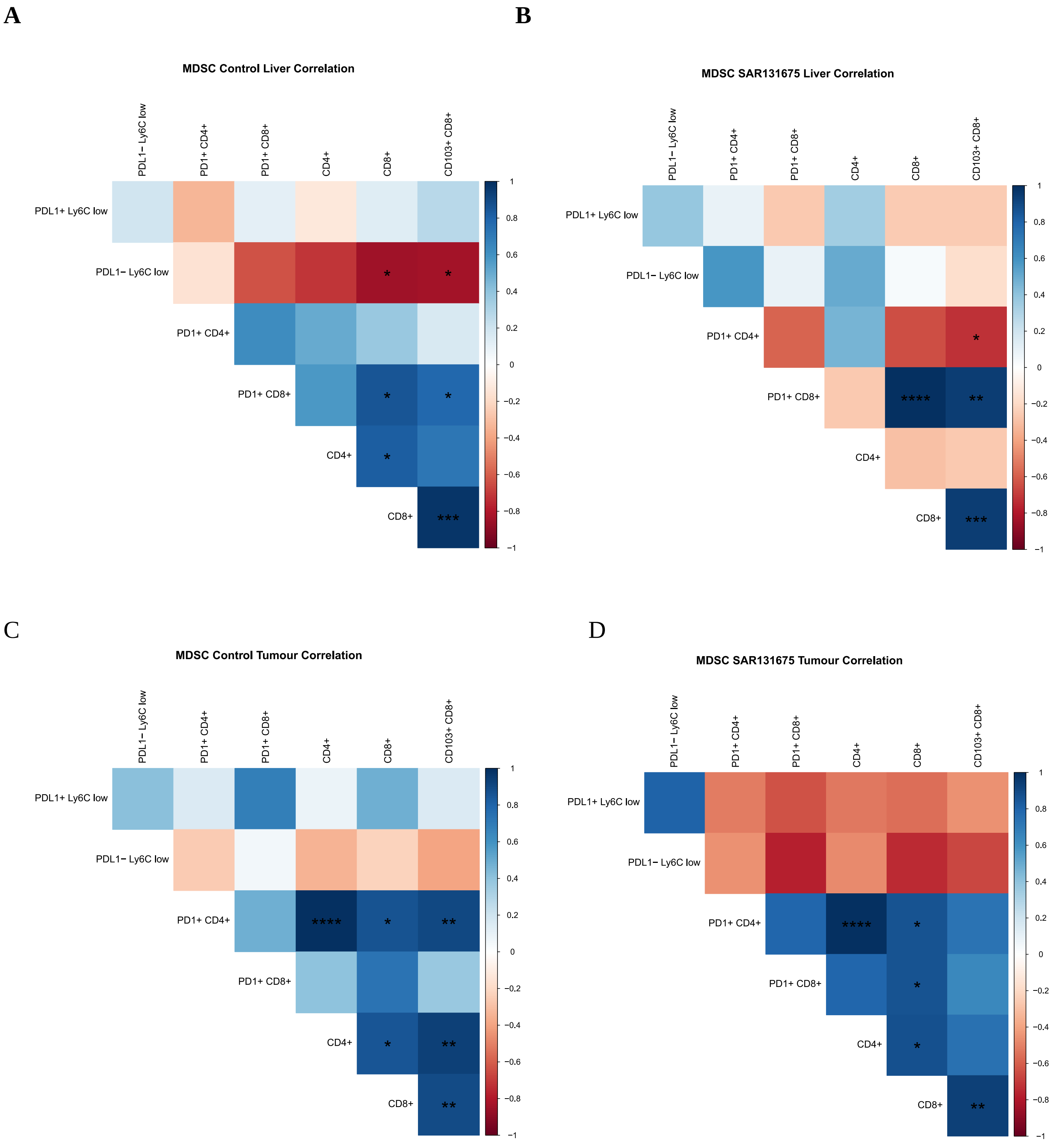

Figure S6 Pearson correlation matrix; Granulocytic MDSC populations
Significant associations indicated by asterix, *p<0.05, **p<0.01, ***p<0.001 and ****p<0.0001.
