# Supplementary material for: SAR131675, a VEGRF3 Inhibitor, Modulates the Immune Response and Reduces the Growth of Colorectal Cancer Liver Metastasis
Source: Cancers (Basel). 2022 May 31;14(11):2715. doi: 10.3390/cancers14112715 (PMC9179346; doi:10.3390/cancers14112715)
Supplement: Supplementary file 1 [file cancers-14-02715-s001.zip › Table S1.pdf]

Table S1 List of antibodies with conditions used for immunohistology

Target Protein	Company	Antibody	Dilution	Linker	Antigen Retrieval	Detection System
**Ki-67**	Thermo Scientific	Rabbit anti-mouse RM-9106-S1	1:200	No linker	Tris buffer	DAB
**CD34**	BIO-RAD	Rat anti-mouse (MCA1825GA)	1:1000	Rabbit anti-rat	Citrate Buffer	DAB
**Podoplanin**	Acris Origene	Syrian hamster anti-mouse (DM3501)	1:500	Rabbit Anti-golden Syrian hamster (PAB10600)	Proteinase K	DAB
**F4/80**	Kindly donated M. Sandrin, Austin Research	Rat anti-mouse	1:200	Rabbit anti-rat	Proteinase K	DAB
**VEGFR-3**	abcam	Rabbit anti-mouse	1:100	No linker	Citrate Buffer	DAB
