# Supplementary material for: SAR131675, a VEGRF3 Inhibitor, Modulates the Immune Response and Reduces the Growth of Colorectal Cancer Liver Metastasis
Source: Cancers (Basel). 2022 May 31;14(11):2715. doi: 10.3390/cancers14112715 (PMC9179346; doi:10.3390/cancers14112715)
Supplement: Supplementary file 1 [file cancers-14-02715-s001.zip › Table S2.pdf]

Table S2. List of antibodies with conditions used for flow cytometry.

Antibody	Fluorophore	Clone	Company	Dilution
**CD45**	BV510	30-f11	BD (563891)	1/1000
**CD3**	PE	145-2C11	BD (553061)	1/200
**CD4**	APC-Cy7	GK1.5	BD (561830)	1/1000
**CD8**	PE-Cy7	53-67	BD (561097)	1/1000
**CD103**	FITC	REA 531	Milteny Biotech (130-108-182)	1/200
**CD11b**	APC-Cy7	M1/70	BD (561039)	1/500
**F4/80**	FITC	REA 126	Miltenyi Biotech (130-117-509)	1/100
**Ly6C**	PE	AL-21	BD (560592)	1/1000
**CD279 (PD-1)**	APC	REA 802	Miltenyi Biotech (131-111-954)	1/200
**CD274 (PD-L1)**	PE-Cy7	M1H1	BD (558017)	1/20
