# Supplementary material for: SAR131675, a VEGRF3 Inhibitor, Modulates the Immune Response and Reduces the Growth of Colorectal Cancer Liver Metastasis
Source: Cancers (Basel). 2022 May 31;14(11):2715. doi: 10.3390/cancers14112715 (PMC9179346; doi:10.3390/cancers14112715)
Supplement: Supplementary file 1 [file cancers-14-02715-s001.zip › Table S3.pdf]

Table S3. T cell subset ratios in Liver and Tumor

	**Ratio**		**CD4: DNT**		**CD8: DNT**	
		subset	total	PD1+	total	PD1+
Liver	Control	Av	0.76	0.20	1.00	0.33
		SD	0.30	0.07	0.21	0.13
	SAR131675	Av	0.73	0.24	0.76	0.24
		SD	0.08	0.06	0.17	0.09
		p value	0.812	0.224	0.041	0.142
Tumor	Control	Av	0.21	0.06	0.55	0.43
		SD	0.09	0.04	0.29	0.39
	SAR131675	Av	0.21	0.05	0.72	0.27
		SD	0.06	0.01	0.24	0.09
		p value	0.9889	0.5728	0.3182	0.4409
